# Aerobic Exercise for Upper Limb Function in a Patient With Severe Paralysis With Subacute Stroke: A Case Report

**DOI:** 10.7759/cureus.39502

**Published:** 2023-05-25

**Authors:** Ayana Kato, Hiroyuki Hayashi

**Affiliations:** 1 Graduate School of Health Care Studies, Seijoh University, Tokai, JPN; 2 Department of Rehabilitation, Tokai Memorial Hospital, Kasugai, JPN; 3 Department of Rehabilitation and Care, Seijoh University, Tokai, JPN

**Keywords:** post-stroke, occupational therapy program, upper limb rehabilitation, aerobic exercise, physical medicine and rehabilitation

## Abstract

Little is known about the effectiveness of aerobic exercise for upper limb function in patients with severe paralysis. We introduced aerobic exercise to improve upper limb function in a patient approximately three months after stroke onset. A 24-year-old woman presented with occlusion of the right internal carotid artery. We introduced high-dose self-rehabilitation for upper limb function, comprising daily three-hour self-rehabilitation sessions for 25 days, in addition to occupational therapy. After the self-rehabilitation phase, we added daily 30-minute aerobic exercise sessions for 25 days, totaling 25 sessions, on a recumbent stationary cycle ergometer. At the start of the aerobic exercise, the assessment scores were as follows: Fugl-Meyer Assessment for upper extremity (FMA-UE), 22/66; Motricity Index (MI), 48; and Motor Activity Log (MAL) for the amount of use (AOU) and quality of movement (QOM), 1.3 and 1.1, respectively. After the 25 aerobic exercise sessions, the assessment scores were as follows: FMA-UE, 32; MI, 61; and MAL for AOU and QOM, 1.6 and 1.3, respectively. The analysis using the percentage of non-overlapping corrected data showed that aerobic exercise was more effective than the self-rehabilitation sessions alone in both the FMA-UE and MI scores. Although future studies should investigate the effects of aerobic exercise in a larger number of patients, improving upper limb function may be facilitated by incorporating aerobic exercise.

## Introduction

Upper limb dysfunction after stroke causes difficulty with activities of daily living (ADLs) and other important activities. Hence, the approach to upper limb dysfunction is common in stroke rehabilitation. Interventions that are supported for the recovery of upper limb function in stroke rehabilitation include task-specific training, constraint-induced movement therapy (CIMT), and the use of biofeedback [1]. However, the severity of upper limb paralysis may limit therapeutic applications, such as CIMT [2], and other interventions may need to be considered for such patients. A previous study [3] of patients with chronic stroke reported that one session of treadmill exercise for 20 minutes improved the action research arm test score from 40 to 42 points. Moreover, a case report [4] on chronic stroke reported that performing aerobic exercise three times a week for eight weeks improved the score of the Fugl-Meyer Assessment for upper extremity (FMA-UE) from 35 to 55. Thus, aerobic exercise may be a useful intervention to improve paralyzed upper limb function. However, despite limited interventions for severely paralyzed upper limbs, little is known about the effectiveness of aerobic exercise on upper limb function in patients with severe paralysis. In addition, there are few reports of studies that introduced aerobic exercise to improve upper limb function in rehabilitation in the subacute phase. Herein, we present the practice of aerobic exercise and recovery of upper limb function in a patient with a severely paralyzed upper limb approximately three months after stroke onset.

## Case presentation

Written informed consent was obtained from the patient for the publication of this case report. The patient was a 24-year-old, right-handed woman with left hemiparesis. She was transported to a hospital by ambulance after a severe headache and decreased consciousness (Japan Coma Scale I-2). Computed tomography and magnetic resonance imaging revealed occlusion of the right internal carotid artery. The patient was prescribed antiplatelet therapy. The patient received treatment and rehabilitation at an acute care hospital and was admitted to the rehabilitation ward of our hospital on day 44 after cerebral infarction onset. At the time of hospitalization at our rehabilitation ward, the FMA-UE score consisting of shoulder, elbow, forearm, wrist, and hand movements including grasp, coordinated movements, and reflex activity was 18/66, indicating severe paralysis, based on the cutoff for "severe" (0-28) [5]. The Motricity Index (MI) score, used to assess muscle strength by examining one movement on the pinch grip, elbow flexion, and shoulder abduction, was 30. The Motor Activity Log (MAL) scores for the amount of use (AOU) and quality of movement (QOM) were 0 and 0, respectively. The passive ranges of joint motion of the shoulder, elbow, forearm, wrist, and fingers were full. The Functional Independence Measure score was 99 points. Based on the initial evaluation, the patient received occupational therapy (OT) daily, including ADL practice; treatment for the paralyzed upper limb, including functional electrical stimulation; wiping, involving the non-paralytic upper limb; mirror therapy; and robotic-assisted training. Immediately after the OT, ADLs improved, except for walking; however, there was little improvement in upper limb function.

OT + self-rehabilitation for upper limb function

The OT session focused on improving upper limb function. OT included repetitive, task-oriented training for upper limb function. Moreover, we introduced high-dose self-rehabilitation for upper limb function consisting of daily three-hour self-rehabilitation sessions for 25 days in addition to OT. Self-rehabilitation included functional electrical stimulation, wiping, mirror therapy, robotic-assisted training, and muscle strengthening for the paralyzed upper limb. We defined phase A as the period of OT and self-rehabilitation. At the start of phase A, the FMA-UE score was 18/66, the MI score was 30, and the MAL scores for the AOU and QOM were 0.7 and 0.5, respectively. The FMA-UE and MI scores were assessed twice a week, and the MAL score was assessed weekly. After a total of 25 sessions in phase A, the FMA-UE score was 22, the MI score was 43, and the MAL scores for the AOU and QOM were 1.1 and 1.0, respectively. However, further improvement in upper limb function was required to reduce the difficulty in using the hands for ADLs. Therefore, we initiated aerobic exercise in addition to self-rehabilitation for the upper limb.

OT + aerobic exercise + self-rehabilitation for upper limb function

We defined phase B as the period of OT, aerobic exercise, and self-rehabilitation. In this phase, the patient performed aerobic exercises immediately before the OT program. At the start of phase B, the FMA-UE score was 22/66; the MI score was 48; and the MAL scores for the AOU and QOM were 1.3 and 1.1, respectively. The patient underwent 30-minute aerobic exercise sessions daily for 25 days, totaling 25 sessions, on a recumbent stationary cycle ergometer. The participant was instructed to maintain her heart rate at 120 bpm as recommended by the American Stroke Association [6]. The heart rate was continuously monitored, and an occupational therapist cued the patient as needed to ensure compliance with the target heart rate of 120 bpm. The patient received OT for upper limb function, forced to task-oriented training, immediately after aerobic exercise. The time for self-rehabilitation for upper limb function continued to be three hours daily. In principle, the content of the self-rehabilitation was the same as in phase A. After a total of 25 sessions of phase B, the FMA-UE score was 32, the MI score was 61, and the MAL scores for the AOU and QOM improved to 1.6 and 1.3, respectively.

Analysis of the effectiveness of aerobic exercise

**Figure 1:**
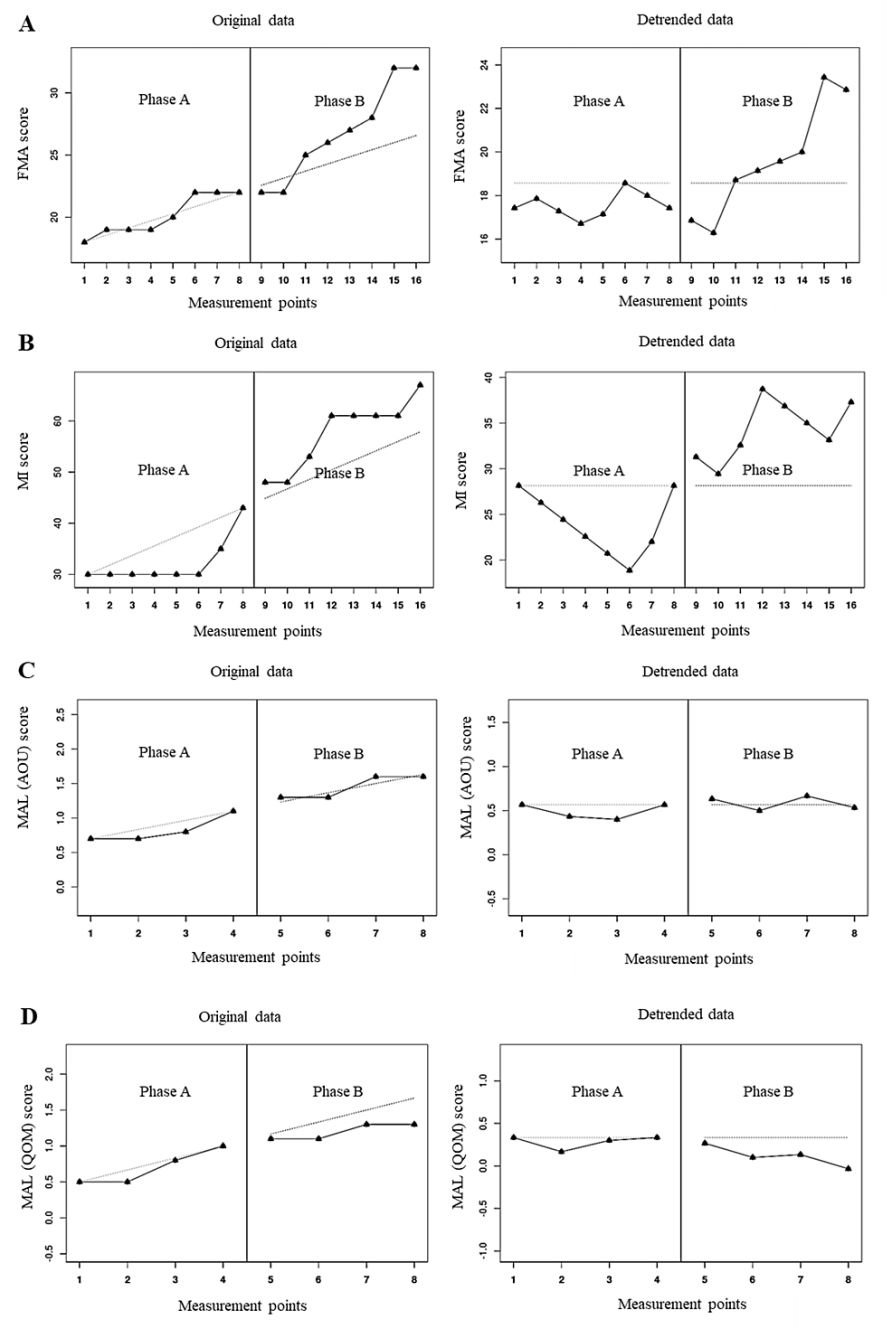
Clinical evaluation in each phase (A) Changes in FMA score in phase A and phase B. (B) Changes in MI score in phase A and phase B. (C) Changes in MAL (AOU) score in phase A and phase B. (D) Changes in MAL (QOM) score in phase A and phase B

We analyzed the percentage of non-overlapping corrected data (PNCD) to confirm the effectiveness of aerobic exercise. PNCD involves a data-correction procedure to remove the baseline trend from the data prior to estimating the change caused by the intervention [7]. Effectiveness was based on the percentage of non-overlapping data between the baseline and intervention phases. A PCND score below 50% was considered ineffective; 50-70%, questionable; 70-90%, effective; and over 90%, very effective [8]. Data analysis was performed using (https://manolov.shinyapps.io/Overlap/) [9]. The results of this analysis demonstrated that the aerobic exercise intervention (phase B) was more effective than the OT and self-rehabilitation intervention (phase A) in the FMA-UE (PNCD, 75%) and MI (PNCD, 100%). However, the aerobic exercise intervention (phase B) demonstrated no greater effectiveness than only the OT and self-rehabilitation intervention (phase A) in the MAL (AOU) (PNCD, 50%) and MAL (QOM) (PNCD, 0%). These data are presented visually in Figure 1.

## Discussion

We introduced aerobic exercise to a patient with severe paralysis three months after stroke onset. In patients with severe upper limb paresis, further recovery of upper limb function should not be expected after 11 weeks [10]. However, our patient continued to recover 12 weeks after the stroke onset. This can be attributed to aerobic exercise since the patient started aerobic exercise 12 weeks after the stroke onset.

A previous study [11] reported that 20 sessions of 25-minute treadmill exercise in subacute patients did not improve the results of the Box and Block test of hand movement, measured as a secondary outcome. However, it is unclear whether upper limb function training was conducted in addition to aerobic exercise in this study. Our patient received OT for upper limb function immediately after aerobic exercise. A previous study [12] showed improvement in upper limb function by conducting task practice immediately after aerobic exercise. Moreover, it has been found that upper limb function improved with 30 minutes of cycling prior to 30 minutes of upper limb training [13]. Performing aerobic exercise immediately before rehabilitation may facilitate improvement in motor function by increasing neuroplasticity [14]. When combined with exercise task practice, this may contribute to improved upper limb function. Moreover, rehabilitation using an ergometer is often performed in the chronic stroke phase [15], and there are limited reports of its application to patients in the subacute stroke phase. Our report was strengthened by applying an ergometer in the subacute phase. The fact that it could also be effective in patients with subacute stroke is valuable in medical insurance systems where the length of stay is limited. On the other hand, the aerobic exercise intervention showed no effect on MAL. Although we conducted repetitive task-oriented training for upper limb function, reaching out and handling an object is difficult for patients with severe paralysis. Aerobic exercise promotes motor learning [14], which may be reflected in the increased use of the paralyzed hand in ADLs when sufficient task-oriented training is performed.

This case report had a limitation. High-intensity aerobic exercise may be effective in functional recovery after stroke [16]. In our patient, the heart rate during exercise was set at 120 bpm, which may have resulted in lower exercise intensity.

## Conclusions

We introduced aerobic exercise to a patient with severe paralysis. After the introduction of aerobic exercise, upper limb function showed improvement. Although future studies should investigate the effects of aerobic exercise in a larger number of patients, an improvement in upper limb function may be expected by incorporating aerobic exercise before upper limb function training.
